# Supplementary material for: DNA Wrapping by a tetrameric bacterial histone
Source: Nat Commun. 2025 Dec 11;16:11108. doi: 10.1038/s41467-025-67425-w (PMC12701072; doi:10.1038/s41467-025-67425-w)
Supplement: Supplementary file 1 — Supplementary Information [file 41467_2025_67425_MOESM1_ESM.pdf]

Supplementary information of

DNA Wrapping by a Tetrameric Bacterial Histone

Yimin Hu[a], Samuel Schwab[b,#], Kaiyu Qiu[a,#], Yunsen Zhang[c], Kerstin Bär[a], Heidi Reichle[a], Aurora Panzera[d], Andrei N. Lupas[a], Marcus D. Hartmann[a,e], Remus T. Dame[b], Vikram Alva[a,*], Birte Hernandez Alvarez[a,*]

[a] Max Planck Institute for Biology Tübingen, Department of Protein Evolution, Tübingen, Germany

[b] Leiden Institute of Chemistry, Leiden University, Leiden, The Netherlands; Centre for Microbial Cell Biology, Leiden University, Leiden, The Netherlands; Centre for Interdisciplinary Genome Research, Leiden University, Leiden, The Netherlands

[c] Theoretical and Computational Biophysics Group, Beckman Institute for Advanced Science and Technology, Center of Biophysics and Quantitative Biology, University of Illinois Urbana-Champaign, Urbana, United States

[d] Max Planck Institute for Biology Tübingen, BioOptics Facility, Tübingen, Germany

[e] Interfaculty Institute of Biochemistry, University of Tübingen, Tübingen, Germany

[#] These authors contributed equally.
* These authors jointly supervised this work.

E-mail: vikram.alva@tuebingen.mpg.de or birte.hernandez@tuebingen.mpg.de

Table of Contents:

Section	Pages
Supplementary Methods	3–8
Supplementary Tables 1–4	9–12
Supplementary Figures 1–21	13–33
Availability of Supplementary Data	34
Supplementary References	35–36

Supplementary Methods

Crystallization, data collection, and structure determination

Crystallization trials were set up by mixing 300 nL of protein with 300 nL of reservoir solution in 96-well sitting-drop vapor-diffusion plates using commercially available screens with reservoir volumes of 100 µL. Without DNA, HLp was prepared at 11 mg/mL in 20 mM Tris, pH 8.0, and 150 mM NaCl. The best diffracting crystals were obtained with a reservoir solution containing 0.1 M citric acid, pH 5.0, and 3.15 M ammonium sulfate. For co-crystallization of HLp and DNA, 500 µM HLp was mixed with 500 µM 30-bp-GC40 dsDNA (Supplementary Table 1) and incubated at 37 °C for 10 min, after which aggregates were removed by centrifugation. The conditions under which the crystals used for structure determination grew were 0.1 M HEPES, pH 7.5 and 25% PEG 6000 for HLp-DNA_1, and the Morpheus condition G12 [0.1 M carboxylic acids, 0.1 M Morpheus Buffer System 3, pH 8.5 and 50% (V/V) Morpheus Precipitant Mix 4] for HLp-DNA_2. For cryo-protection, the crystals were transferred to droplets of their reservoir solution spiked with 30% glycerol (free HLp) or 15% PEG 400 (HLp-DNA_1), loop-mounted, and flash frozen in liquid nitrogen. Data were collected at beamline X10SA of the Swiss Light Source (Villigen, Switzerland) at 100 K, using an EIGER X 16M hybrid pixel detector (Dectris, Ltd.). Data were reduced, processed, and scaled using XDS (package 2024)[1]. Due to pronounced anisotropy, diffraction data for HLp-DNA_2 were submitted to the STARANISO (version 2.4.19) server for ellipsoidal truncation and anisotropic scaling, following the unmerged data protocol[2].

The structure of free HLp was solved by molecular replacement (MR) using MOLREP (version 11.7.02) and an AlphaFold2 prediction as a search model, locating one HLp dimer in the asymmetric unit (ASU)[3-5]. The DNA-bound structures were solved by MR using MOLREP (version 11.7.02) and the refined free HLp coordinates as a search model, locating an HLp dimer and 16 bp of dsDNA in the ASU for HLp-DNA_1, and a monomer and 15 nucleotides of ssDNA in the ASU for HLp-DNA_2. The three structures were modeled, refined, and finalized in cycles of manual modeling in Coot (version 0.9) and refinement with REFMAC5 (version 5.8.0266)[6,7]. Data processing and refinement statistics are given in Supplementary Table 3. The coordinates and structure factors have been deposited in the PDB under the accession numbers 9QT0 (free HLp), 9QT1 (HLp-DNA_1), and 9QT2 (HLp-DNA_2). All structures were visualized using PyMOL (The PyMOL Molecular Graphics System, version 3.1.1, Schrödinger, LLC.).

To compare tetramers formed by FtF and nucleosomal histones, the angle between the non-contacting α2 helices of adjacent dimers was defined as the dimer-dimer angle and calculated using the provided Python script (Supplementary code).

Molecular dynamics (MD) simulation

We performed all-atom molecular dynamics (MD) simulations in GROMACS (version 2023.2) to study two possible topologies of the HLp-DNA complexes formed by bridging or wrapping[8]. The DNAs in the HLp-DNA_1 and HLp-DNA_2 structures were extended through crystal symmetry. The obtained structures were superimposed based on the central HLp, the excess DNA fragments were trimmed up to the overlap point, and then the

remaining DNA fragments were connected to obtain the two initial structures of HLp-DNA complexes (Fig. 5; Supplementary Figs. 9, 10). Two initial models were constructed for each binding mode (wrapping and bridging) based on the HLp-DNA_1 and HLp-DNA_2 structures. One representative model for each binding mode was pursued further. The constructed models were then preprocessed using PDBFixer (version 1.10, https://github.com/openmm/pdbfixer) to add missing hydrogen atoms and adjust chain configurations as necessary. The CHARMM all-atom force field (CHARMM36m) was applied to parameterize and describe both intra- and intermolecular interactions between the protein and DNA[9]. Ion parameters and the TIP3P water model were selected following the CHARMM36m recommendations[10]. Each system was solvated in a sufficiently large cubic water box to ensure a minimum water layer of 1.5 nm surrounding the protein-DNA complex in all directions. Sodium and chloride ions were added to neutralize the system. Periodic boundary conditions were imposed to eliminate edge effects, enabling the simulation to be conducted under an infinite solvent environment.

Each system underwent a multistage process of energy minimization and equilibration before production simulation. First, a harmonic positional restraint of 1000 kJ/(mol·nm^2) was applied to the backbones of both proteins and DNAs to relax the distributions of water molecules and ions. The system was then subjected to 5000 steps of conjugate gradient and steepest descent minimization until the maximum force on any atom dropped below a predefined threshold of 1000 kJ/(mol·nm^2). Following energy minimization, each complex system was equilibrated in multiple stages using the canonical (NVT) and isothermal-isobaric (NPT) ensembles. During the NVT phase, the system was gradually heated to 303.15 K with a V-rescale thermostat[11]. In the NPT phase, density equilibration was achieved at a constant pressure of 1 atm using a Parrinello-Rahman barostat[12]. Once equilibration was complete, a 1 μs production simulation was conducted on the processed protein-DNA complex, with the integration carried out using the leap-frog algorithm at a timestep of 2 fs. Meanwhile, a 1.2 nm cutoff was set for van der Waals and electrostatic potentials to calculate the short-range interaction, and the Particle Mesh Ewald method was employed to compute the long-range interaction[13]. To enhance computational efficiency, the LINCS algorithm was employed to constrain hydrogen bond lengths in water molecules and the backbones of both proteins and DNAs[14]. Two independent 1 μs simulations were performed for each system to ensure the robustness of the results (Supplementary Movies 1 and 2).

Both simulations exhibited highly similar behaviors in either the wrapping or the bridging model (Fig. 5b; Supplementary Fig. 11); therefore, the analysis was conducted on one representative trajectory for each system. To assess global structural stability, we calculated the root mean square deviation (RMSD) of the DNA backbone relative to the initial structure. Binding fluctuations were evaluated by determining the difference between the contact number of each frame and the average contact number over all captured frames. A protein-DNA contact was defined and counted if any protein heavy atom was within 0.4 nm of a DNA heavy atom. Hydrogen bonds were identified with a distance cutoff of 0.3 nm for the donor-to-acceptor (D-A) distance and an angular cutoff of 150° for the donor-hydrogen-acceptor (D-H-A) angle. To further study the binding dynamics of the local interaction sites, we calculated the binding contact number for each residue throughout the simulation and manually selected residues to define four binding sites (Fig. 5c; Supplementary Table 4).

To quantify dihedral angle fluctuation during simulations (Supplementary Fig. 13a), we defined two dimers corresponding to residues 1–116 and 117–232 using Cα coordinates. The best-fit planes were determined using singular value decomposition, and the dihedral angle between the dimers was computed from the dot product of the resulting plane normals.

Free-energy landscapes (FELs) were constructed from simulation trajectories using two biophysically meaningful collective variables: (i) the root-mean-square deviation (RMSD) of the DNA phosphate (P) atoms relative to the initial reference conformation, and (ii) the radius of gyration (Rg) of the DNA. The trajectories were preprocessed to remove periodic boundary artefacts and truncated to exclude equilibration frames. The RMSD and Rg values were then computed for each saved frame and binned onto a two-dimensional grid. FELs were reconstructed according to $F = -k_B T ln P + C$ at T = 300 K, where C is an arbitrary constant. FEL visualization was performed in Python (version 3.8.5) using Matplotlib package (version 3.6.2)[15,16].

To further characterize and understand the binding dynamics of the proposed wrapping model, an unwrapping simulation was performed in the framework of a two-step steered molecular dynamics (SMD)[17], with the constructed wrapping model (Supplementary Fig 10) as the starting structure. The first SMD simulation targeted the dissociation of the B-site 2, followed by a second SMD simulation that used the last snapshot from the B-site dissociation as the starting structure to investigate the unbinding of the A-site. In the SMD setup, a reaction coordinate was defined by connecting the center of mass of the relevant DNA regions (residues 1–12) to that of the protein, ensuring that the applied force was directed along an axis expected to promote the unwrapping process. A pulling velocity of 0.002 nm/ps and a harmonic spring constant of 500 kJ/(mol·nm²) were selected to balance the computational feasibility with the need to capture quasi-equilibrium behavior. During the production simulation of SMD, force and extension data were recorded at regular intervals to allow integration of the force over distance and estimation of the work required for unwrapping. Simultaneously, the temporal evolution of protein-DNA contacts was monitored using a 0.4 nm cutoff to pinpoint specific events where the DNA disengaged from the binding interface. Once both B-site 2 and A-site 2 were dissociated, we integrated the force-distance curves obtained from the SMD simulation to generate dissociation energy profiles. All simulations were analyzed using VMD (version 1.9.4) and the MDAnalysis package (version 2.8.0), with Matplotlib (version 3.6.2) and Seaborn (version 0.12.2) employed for visualization[15,18-20].

DNA binding assays

Electrophoretic mobility shift assay (EMSA)

The 80-bp dsDNA fragment, 30-bp dsDNA fragments of various GC content (30%, 40%, 50%, and 60%), and the GeneRuler 1 kb Ladder (ThermoFisher Scientific) were used for *in vitro* DNA binding tests. For comparative DNA binding analysis, the proteins were mixed with the annealed dsDNA fragments at the indicated molar ratios in a binding buffer containing 25 mM Tris, pH 8.0, 50 mM NaCl, and 50 mM KCl. Following incubation at 37 ºC for 10 min, the samples were separated on a 6% DNA retardation gel (ThermoFisher Scientific). To analyze the binding of HLp and HMfB to the GeneRuler 1 kb Ladder, the proteins and the DNA were

mixed at the indicated mass ratios in the binding buffer and incubated at 37 ºC for 10 min. Samples were separated on a 1% agarose gel, and the DNA was visualized using SYBR Gold nucleic acid gel stain (ThermoFisher Scientific) and imaged using the Fusion SL imaging system (Vilber). All EMSAs were performed in 2–3 independent replicates using separate protein preparations.

Micrococcal nuclease (MNase) digestion assay—600-bp-GC40 DNA fragment

The 600-bp-GC40 DNA fragment was amplified using primers pET-600-bp-GC40-F and pET-600-bp-GC40-R with pETHis1a[21] as a template, followed by purification using the QIAquick PCR Purification Kit (QIAGEN). For HLp-DNA complex formation, 720 ng of HLp was mixed with 900 ng of 600-bp-GC40 DNA in MNase digestion buffer (New England Biolabs) and incubated at 37 ºC for 10 min. The indicated amount of MNase (New England Biolabs) was added, yielding a final assay buffer composition of 10 mM Tris (pH 8.0), 75 mM NaCl, and 1 mM $CaCl_2$. Digestion was performed in an 80 µL reaction volume at 37 °C for 10 min. As a positive control, HMfB (540 ng) was preincubated with 600-bp-GC40 DNA (900 ng) and treated with the indicated amounts of MNase at 37 ºC for 15 min. EDTA and SDS were added to final concentrations of 95 mM and 0.5%, respectively, to stop the reaction. DNA fragments protected by bound proteins were purified by phenol/chloroform extraction followed by ethanol precipitation. The purified DNA fraction was dissolved in 5 µL of water and separated on a 10% Novex TBE gel (ThermoFisher Scientific). The gel was stained with SYBR Gold nucleic acid gel stain (ThermoFisher Scientific) and imaged using the Gel Doc XR+ imaging system (Bio-Rad Laboratories). All experiments were performed at least in triplicate, and the length of the selected DNA bands was calculated using Image Lab 6.1 (Bio-Rad Laboratories). For better visualization, the image background was subtracted using the rolling-ball algorithm with a radius of 50 pixels in FIJI (version 2.3.0, https://fiji.sc/)[22].

Micrococcal nuclease (MNase) digestion assay—E. coli

E. coli BL21(DE3) and *E. coli* Mutant56(DE3)[23], transformed with plasmids pET-30a(+) encoding HLp and pET-28a(+) encoding HMfB, respectively, were grown in LB medium. Protein expression was induced with 1 mM IPTG, while uninduced cultures served as negative controls.

The chromatinization ability of HLp *in vivo* was analyzed following published protocols with minor modifications[24,25]. Cells from 10-mL cultures were pelleted (7000 × *g*, 10 min, 4 °C), washed, and resuspended in PBS. Fixation was performed with 1% formaldehyde in PBS for 10 min and quenched with 145 mM glycine for 10 min. Cells were pelleted, washed twice with cold PBS, and resuspended in lysozyme buffer (120 mM Tris, pH 8.0, 50 mM EDTA, 4 mg/mL lysozyme). After incubation at 37 °C for 40 min with gentle agitation, protoplasts were collected by centrifugation and resuspended in 500 µL lysis buffer [10 mM Tris, pH 8.0, 10 mM NaCl, 3 mM $MgCl_2$, 0.5% NP-40, protease inhibitor mix (cOmplete™, EDTA-free, Roche), 0.15 mM spermine, 0.5 mM spermidine]. Samples were incubated on ice for 30 min, pelleted, and washed with -CA buffer (10 mM Tris, pH 8.0, 15 mM NaCl, 60 mM KCl, protease inhibitor mix, 0.15 mM spermine, 0.5 mM spermidine). Pellets were resuspended in +CA buffer (-CA buffer supplemented with 1 mM $CaCl_2$).

Aliquots (25 µL) were digested with the indicated amounts of MNase (NEB) in 50 µL

reactions at 37 °C for 20 min. Reactions were stopped with 100 mM EDTA and 10 mM EGTA. Samples were then treated with proteinase K (150 µg/mL, ThermoFisher Scientific) and 1% SDS and incubated overnight at 55 °C. DNA protected by HLp was purified by two rounds of phenol/chloroform extraction with RNase A treatment (100 µg/mL, 37 °C, 2 h, ThermoFisher Scientific) between extractions, followed by chloroform extraction and ethanol precipitation. DNA pellets were washed with cold 70% ethanol, resuspended in 40 µL sterile water, and resolved on 10% Novex TBE gels (ThermoFisher Scientific). Gels were stained with SYBR Gold (ThermoFisher Scientific) and imaged using a Gel Doc XR+ system (Bio-Rad).

All experiments were conducted in triplicate. Band sizes were determined using Image Lab 6.1 (Bio-Rad). Background subtraction was applied with the rolling-ball algorithm (radius = 50 pixels) in FIJI v2.3.0 (https://fiji.sc/)[22]. DNA fragment sizes were determined by densitometric analysis, using the molecular weight marker M2 (GeneRuler Ultra Low Range DNA Ladder, ThermoFisher Scientific) as the standard.

Tethered particle motion (TPM) assay

TPM experiments were performed as previously described in 50 mM Tris, pH 7.0, and 75 mM KCl[26]. A standard deviation cutoff of 8% and an anisotropic ratio cutoff of 1.3 were used to select single-tethered beads. Measurements at each HLp concentration were done in triplicate. Means and standard deviations of the individual measurement series for each HLp concentration were calculated by maximum likelihood estimation, assuming a normal distribution. Outliers with a robust Z-score >3 or <-3 were not considered for fitting. The "line to guide the eye" was generated by fitting the means to a logistic function. A custom Python script was used to fit and plot the TPM data[16]. For plotting, the means of the three individual measurements were averaged for each measured concentration, and the standard deviations were error-propagated ($Std(\overline{X}) = \sqrt[2]{\frac{\sum_{i=1}^{n} Var(X_i)}{n^2}}$).

DNA topology assay

Plasmid pUC19 was purified from *E. coli* Top10 using NucleoSpin Plasmid QuickPure Kit (Macherey-Nagel). For topological relaxation, the plasmid pUC19 was treated with the nicking endonuclease Nb.BsrDI (New England BioLabs), followed by ligation with T4 DNA ligase (ThermoFisher Scientific). Plasmid relaxation was verified by agarose gel electrophoresis. 200 ng of relaxed pUC19 was mixed with HLp or HMfB at indicated protein-to-DNA mass ratios in assay buffer containing 10 mM Tris, pH 7.0, and 75 mM NaCl) and incubated at room temperature for 30 min. Then, 1 U of Topoisomerase I (ThermoFisher Scientific) was added, and the reaction mixture was incubated at 37 ºC for 30 min in a final volume of 50 µL. DNA was purified by phenol/chloroform extraction followed by ethanol precipitation. Purified DNA samples were separated on a 0.8% TAE agarose gel. Plasmids were visualized by staining with SYBR Gold nucleic acid gel stain (ThermoFisher Scientific) and imaged using the Gel Doc XR+ imaging system (Bio-Rad Laboratories).

Ligase-mediated circularization assay

The 240-bp-GC40 DNA fragment was amplified from plasmid pETHis1a using primers pET-240-bp-GC40-Fp and pET-240-bp-GC40-Rp (Supplementary Table 1) and purified using

QIAquick PCR Purification Kit (QIAGEN). 400 ng of DNA was incubated with HMfB and HLp at the indicated protein-to-DNA mass ratios in LMC buffer (10 mM Tris, pH 7.0, 75 mM KCl, and 5% glycerol) in a total reaction volume of 20 μL at room temperature for 30 min. T4 DNA ligase buffer and T4 ligase at a final concentration of 1 U/μL were added in a final reaction volume of 100 μL. A sample not treated with T4 DNA ligase was used as a control. The sample was incubated for 24 h at room temperature, and the DNA was purified by phenol/chloroform extraction and ethanol precipitation. Half of each sample was treated with 1 U of T5 exonuclease (New England BioLabs) in the appropriate buffer at 37 ºC for 1 h. DNA samples were separated on a 2% TAE agarose gel, stained with SYBR Gold nucleic acid gel stain (ThermoFisher Scientific), and imaged using the Gel Doc XR+ imaging system (Bio-Rad Laboratories).

Microscale thermophoresis (MST)

To determine the DNA binding affinity of HLp to the Cy5 labeled 80-bp and 80-bp-GC40 dsDNAs, a dilution series of HLp was prepared in 20 mM Tris, pH 8.0, 150 mM NaCl, and titrated against the dsDNA fragments at a concentration of 10 nM. The HLp-DNA mixtures were incubated at 37 ºC for 10 min and loaded into Monolith NT premium capillaries (MO-K025, NanoTemper Technologies) after centrifugation to remove precipitates. HMfB was used as a positive control and titrated against both DNA fragments at a concentration of 20 nM. HMfB-DNA mixtures were incubated at room temperature for 5 min, centrifuged, and loaded into Monolith NT capillaries (MO-K022, NanoTemper Technologies). All measurements were performed in triplicate at 25 ºC using the Monolith NT 115 instrument with a Nano RED detector and MST power set to medium. MST data were analyzed by fitting them to a K_d model using MO Control V1.6 (NanoTemper Technologies).

Supplementary Tables

Supplementary Table 1

Oligonucleotides used in this work.

Oligo name	Sequence (5' to 3')	Application
30-bp-GC30-F	TTTAAAACGCTTTAAAACGCTTTAAAACGC	EMSA
30-bp-GC30-R	GCGTTTTAAAGCGTTTTAAAGCGTTTTAAA	EMSA
30-bp-GC40-F	TTTAAAGCCGTTTAAAGCCGTTTAAAGCCG	SEC-MALS, EMSA, Crystallization
30-bp-GC40-R	CGGCTTTAAACGGCTTTAAACGGCTTTAAA	SEC-MALS, EMSA, Crystallization
30-bp-GC50-F	TTAAAGCCCGTTAAAGCCCGTTAAAGCCCG	EMSA
30-bp-GC50-R	CGGGCTTTAACGGGCTTTAACGGGCTTTAA	EMSA
30-bp-GC60-F	TTAAGCCCCGTTAAGCCCCGTTAAGCCCCG	EMSA
30-bp-GC60-R	CGGGGCTTAACGGGGCTTAACGGGGCTTAA	EMSA
80-bp-DNA-F	CCGTACTGTCGTCTGCGGCCTTTGATTATCAAT TAAAGCGTTCTACGGCGTTTTTGATCGCTCAA CGTGCGGAGCTAGAT	EMSA
80-bp-DNA-F[Cy5]	[Cyanine5]CCGTACTGTCGTCTGCGGCCTTTGAT TATCAATTAAAGCGTTCTACGGCGTTTTTGATC GCTCAACGTGCGGAGCTAGAT	MST
80-bp-DNA-R	ATCTAGCTCCGCACGTTGAGCGATCAAAAACG CCGTAGAACGCTTTAATTGATAATCAAAGGCC GCAGACGACAGTACGG	EMSA, MST
pET-600-bp-GC40-F	CGCGAATTTTAACAAAATATTAACGTTTACA	MNase digestion
pET-600-bp-GC40-R	ATTCAGGTGAAAATATTGTTGATGCG	MNase digestion
80-bp-GC40-F[Cy5]	[Cyanine5]TTTAAAGCCGTTTAAAGCCGTTTAA AGCCGTTTAAAGCCGTTTAAAGCCGTTTAAAG CCGTTTAAAGCCGTTTAAAGCCG	MST
80-bp-GC40-R	CGGCTTTAAACGGCTTTAAACGGCTTTAAACG GCTTTAAACGGCTTTAAACGGCTTTAAACGGC TTTAAACGGCTTTAAA	MST
685-bp-DNA-F[Biotin]	[Biotin]TTACTTTCACCAGCGTTTCTGGGTGAG CAAAAACAG	TPM
685-bp-DNA-R[DIG]	[DIG]CCAAGTAGCGAAGCGAGCAGGACTGGG CGG	TPM
pET-240-bp-GC40-Fp	[Phos]TGCAATTTATTCATATCAGGATTATCA	Ligase-mediated circularization assay
pET-240-bp-GC40-Rp	[Phos]GCATAAACTTTTGCCATTCTCACC	Ligase-mediated circularization assay

Supplementary Table 2

Binding affinities (K_d) of HLp and HMfB to DNA substrates measured with MST.

Protein	DNA substrate	K_d ± SD* (μM)
HLp	80-bp DNA	3.18 ± 1.06
	80-bp-GC40 DNA	1.24 ± 0.14
HMfB	80-bp DNA	0.58 ± 0.13
	80-bp-GC40 DNA	0.31 ± 0.08

* Average K_d value determined from three independent measurements with standard deviation (SD).

Supplementary Table 3

Data collection and refinement statistics of DNA-free HLp, HLp-DNA_1 and HLp-DNA_2.

	HLp	HLp-DNA_1	HLp-DNA_2
**Data collection**			
Space group	$P3_121$	$P4_12_12$	F222
Cell dimensions			
a, *b*, *c* (Å)	55.70, 55.70, 58.46	67.93, 67.93, 97.90	48.77, 91.61, 100.33
α, β, γ (°)	90, 90, 120	90, 90, 90	90, 90, 90
Resolution range (Å)	48.25-1.30 (1.38-1.30)	43.16-2.10 (2.23-2.10)	39.56-1.90 (2.05-1.90)
Completeness (%)	99.7 (98.3)	99.7 (99.5)	68.4 (19.9) [85.4 (48.4)]
Redundancy	9.43 (5.34)	5.62 (5.53)	7.51 (6.44)
$\langle I/\sigma(I)\rangle$	19.87 (1.14)	11.09 (1.96)	13.86 (1.34)
R_{meas}	0.052 (1.40)	0.081 (0.743)	0.060 (1.534)
**Refinement**			
No. of reflections, working set	24883	13229	5261
No. of reflections, test set	1303	696	918
Final R_{cryst}	0.175	0.250	0.235
Final R_{free}	0.211	0.280	0.288
R.m.s. deviations			
Bonds (Å)	0.0046	0.0068	0.0042
Angles (°)	1.164	1.297	1.173

Values for the outer shell are given in parentheses.
Values for the ellipsoidal completeness are given in square brackets.

Supplementary Table 4

Amino acid residues of the four defined binding sites.

Binding site	Residues (Chain ID-Residue ID)
**A-site 1**	A-MET26, A-THR27, A-SER28, A-GLY29, B-LYS54, B-ARG55, B-THR56, B-THR57, B-VAL58, B-ARG59, C-MET26, C-THR27, C-SER28, C-GLY29, D-LYS54, D-ARG55, D-THR56, D-THR57, D-VAL58, D-ARG59
**A-site 2**	A-LYS54, A-ARG55, A-THR56, A-THR57, A-VAL58, A-ARG59, B-MET26, B-THR27, B-SER28, B-GLY29, C-LYS54, C-ARG55, C-THR56, C-THR57, C-VAL58, C-ARG59, D-MET26, D-THR27, D-SER28, D-GLY29
**B-site 1**	C-ILE11, C-VAL12, C-ALA-13, C-SER14, C-LYS15, C-LYS17, C-LYS21, D-ILE11, D-VAL12, D-ALA-13, D-SER14, D-LYS15, D-LYS17, D-LYS21
**B-site 2**	A-ILE11, A-VAL12, A-ALA-13, A-SER14, A-LYS15, A-LYS17, A-LYS21, B-ILE11, B-VAL12, B-ALA-13, B-SER14, B-LYS15, B-LYS17, B-LYS21

Supplementary figures

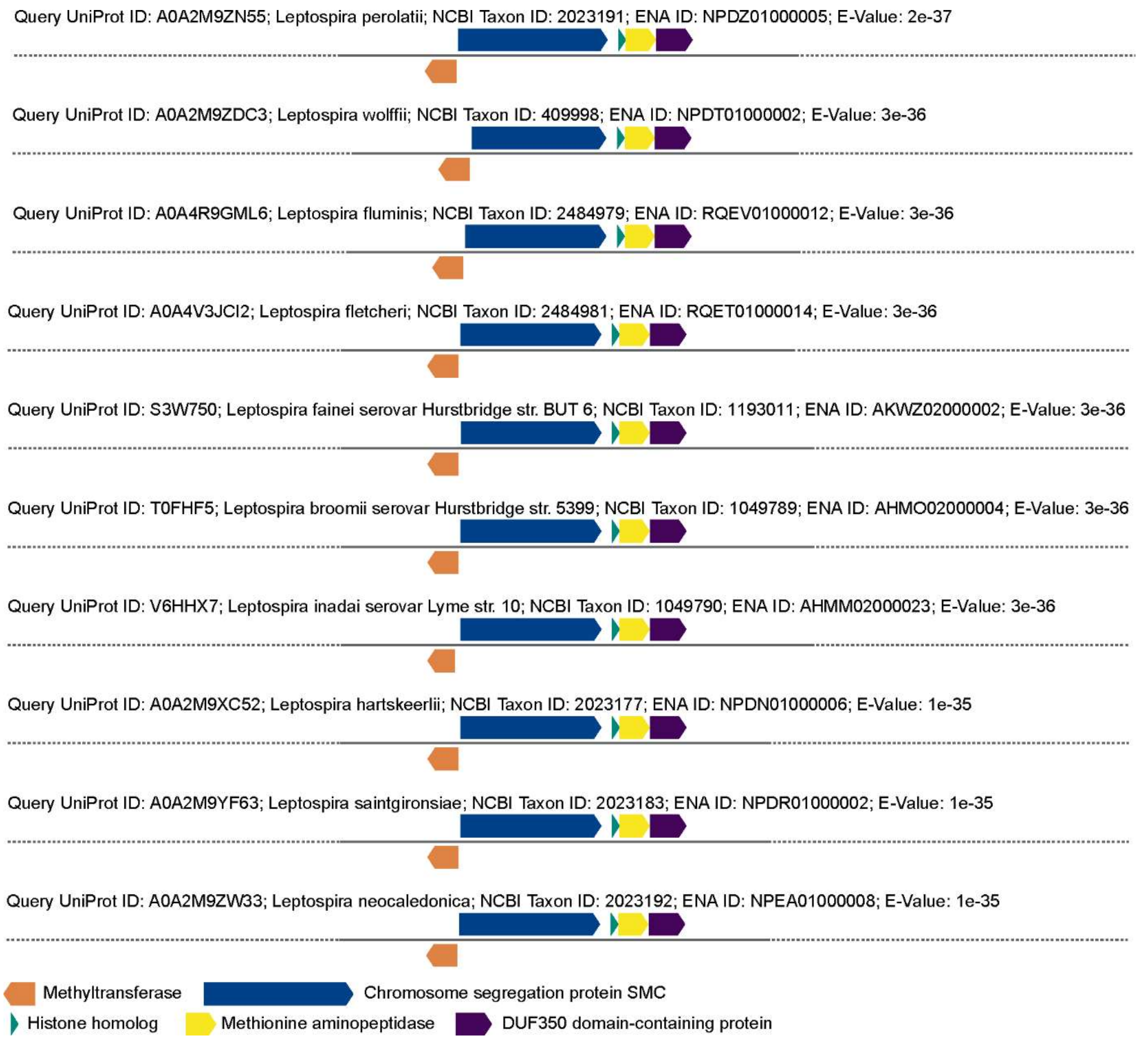

Supplementary Fig. 1

Conserved genomic context of HLp across Leptospira strains. Genomic neighborhood diagrams showing HLp and its homologs in various Leptospira species, illustrating conserved synteny and gene context.

>NPDY01000005.1:141450-141799 Leptospira perolatii strain FH1-B-C1 contig_5, whole genome shotgun sequence
CTTCTGAAAATGATTCCTTTTTTGAGGGTATTCTGGAATGGTATCTTTCTAAGTACTGAGTACCTTCTATAAGAACTTACAGAA
AAGGAGAAAGAGCTCCCCGGCAAAAATTGGAT**ATG**GTACAAAAGAAAAAAACTACAGTTAAGAGGAAGAGTTCC**ATG**GCTCAAA
ACGCTGAAAAGGATACCCTCATCGTCGCAAGTAAGGTGAAAGCCTATATCAAATCCAAAGGGTTCATGACTTCTGGGGATGCAG
TCGATGGTTTGAATGAAAAGTTGTACGCATTAATCGATGATGCGTTAAAGCGCACTGAGTCCAACAAACGGACTACGGTTCGCC
CAACCGACTTCTAA

>WP_207761458.1 hypothetical protein [Leptospira perolatii]
MVQKKKTTVKRKSS**M**AQNAEKDTLIVASKVKAYIKSKGFMTSGDAVDGLNEKLYALIDDALKRTESNKRT
TVRPTDF

>PJZ70190.1 hypothetical protein CH360_07900 [Leptospira perolatii]
MAQNAEKDTLIVASKVKAYIKSKGFMTSGDAVDGLNEKLYALIDDALKRTESNKRTTVRPTDF

Supplementary Fig. 2
DNA and protein sequences of HLp. Nucleotide and amino acid sequences of HLp. The start codon of the longer *hlp* gene and its corresponding methionine are colored green, while those of the shorter gene variant are highlighted in orange. The Shine-Dalgarno sequence is highlighted in yellow.

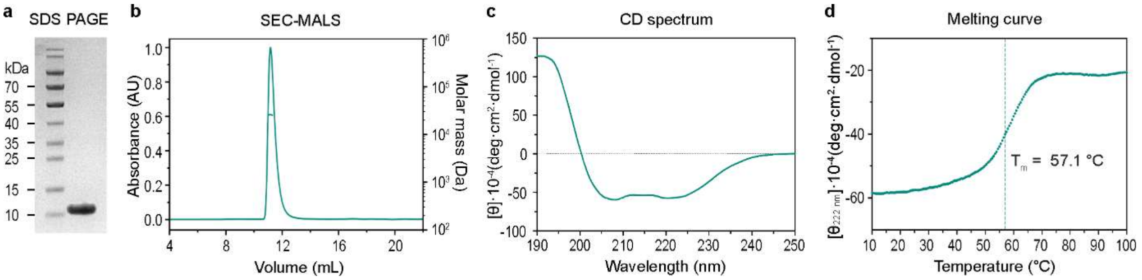

Supplementary Fig. 3

Biophysical characterization of HLp in terms of purity, stability, and oligomeric state. a SDS-PAGE showing purified HLp. **b** SEC-MALS analysis of HLp showing its tetrameric state. **c** Single CD spectrum of HLp. **d** Thermal melting curve of HLp measured with CD spectroscopy at a wavelength of 222 nm.

All source data and original uncropped gel images are provided as a Source Data file.

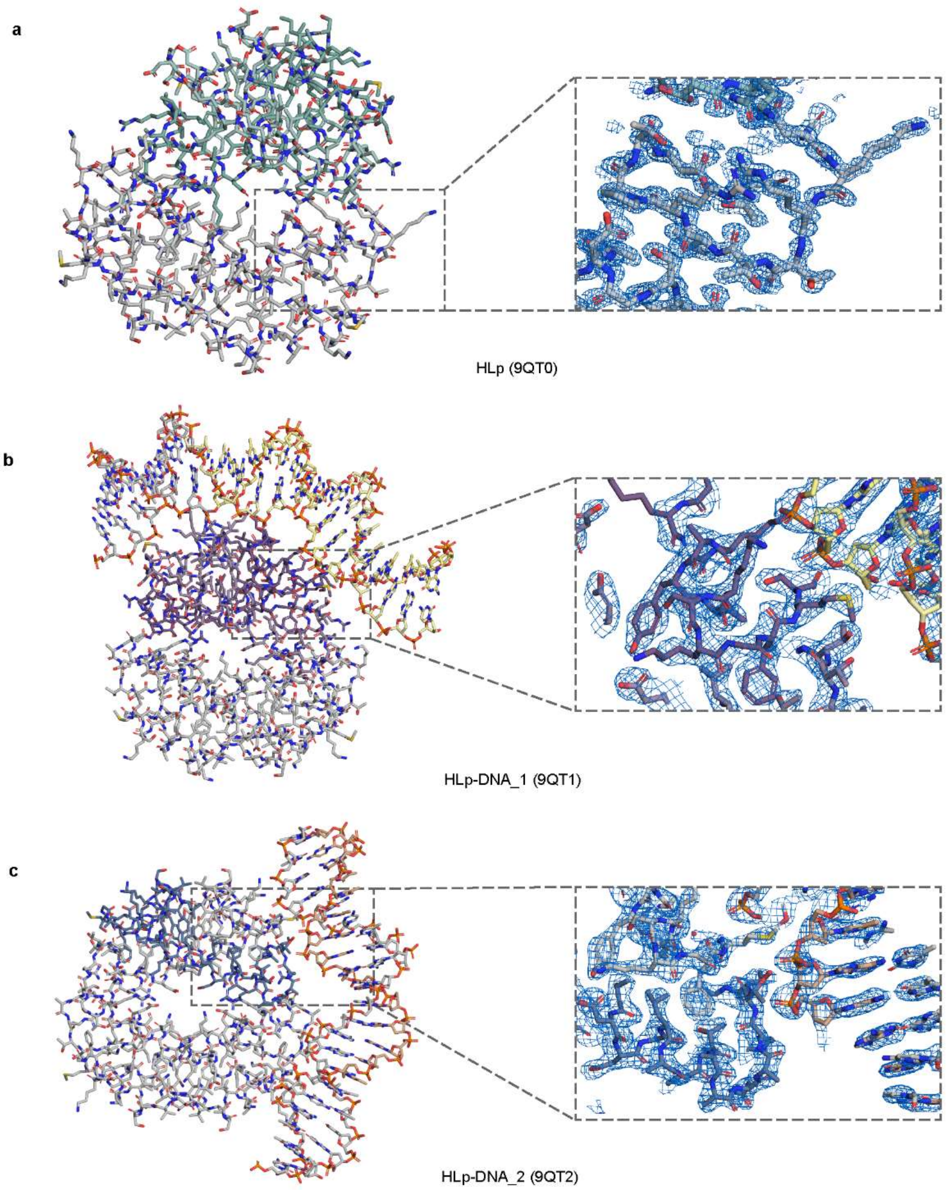

Supplementary Fig. 4

Crystal structures with representative electron density maps. HLp (**a**), HLp-DNA_1 (**b**), and HLp-DNA_2 (**c**) are shown in stick representation, with corresponding 2Fo-Fc electron density maps contoured at 1.5 σ highlighted in close-up views. The protein within the asymmetric unit is shown in color, symmetry mates in gray.

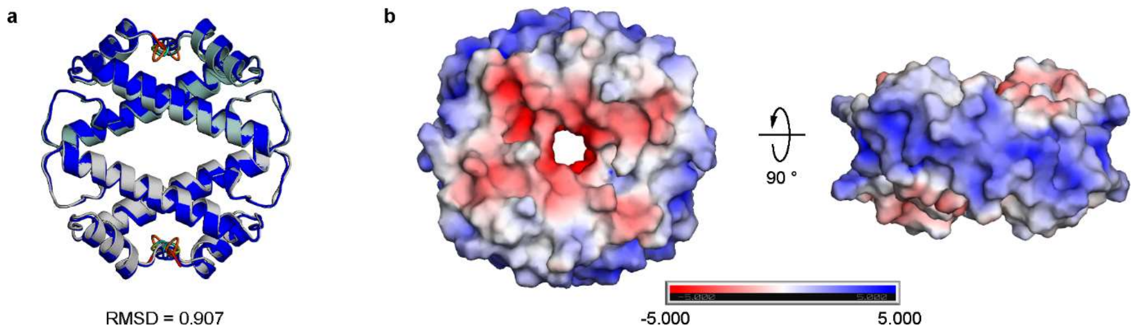

Supplementary Fig. 5

Analysis of the HLp crystal structure. a Superposition of the crystal structure of the HLp tetramer with its AlphaFold2 prediction, showing an RMSD of 0.907 Å. **b** APBS (Advanced Poisson-Boltzmann Solver) electrostatic analysis of the HLp tetrameric structure reveals a continuous, positively charged surface encircling the entire tetramer.

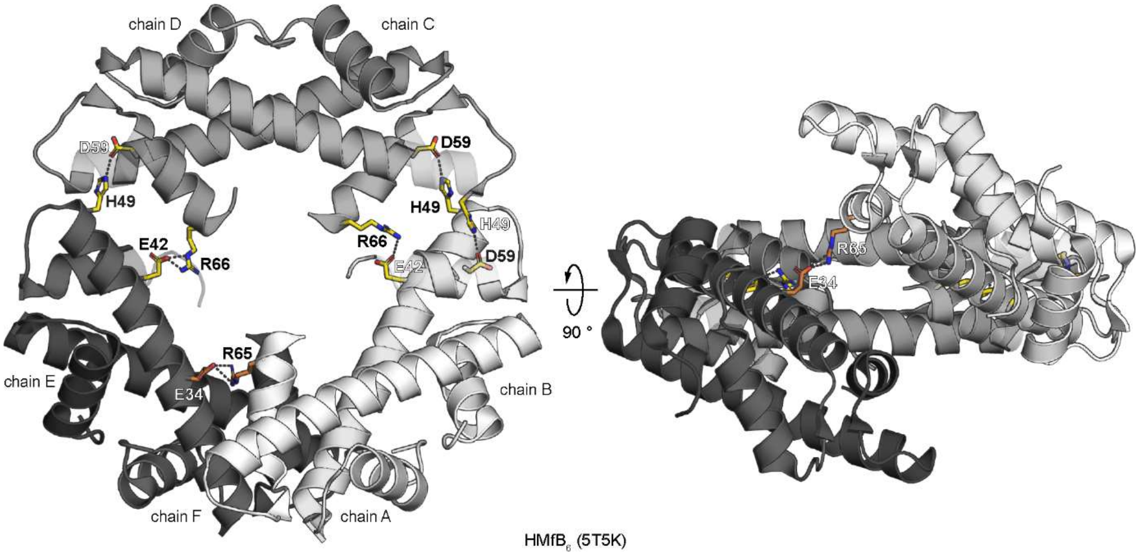

Supplementary Fig. 6
Structure of three assembled HMfB dimers. Crystal structure of three HMfB dimers in spiral arrangement (PDB: 5T5K) in cartoon representation. Residues involved in oligomerization are shown as sticks and the salt bridges formed between HMfB dimers are indicated as dashed lines.

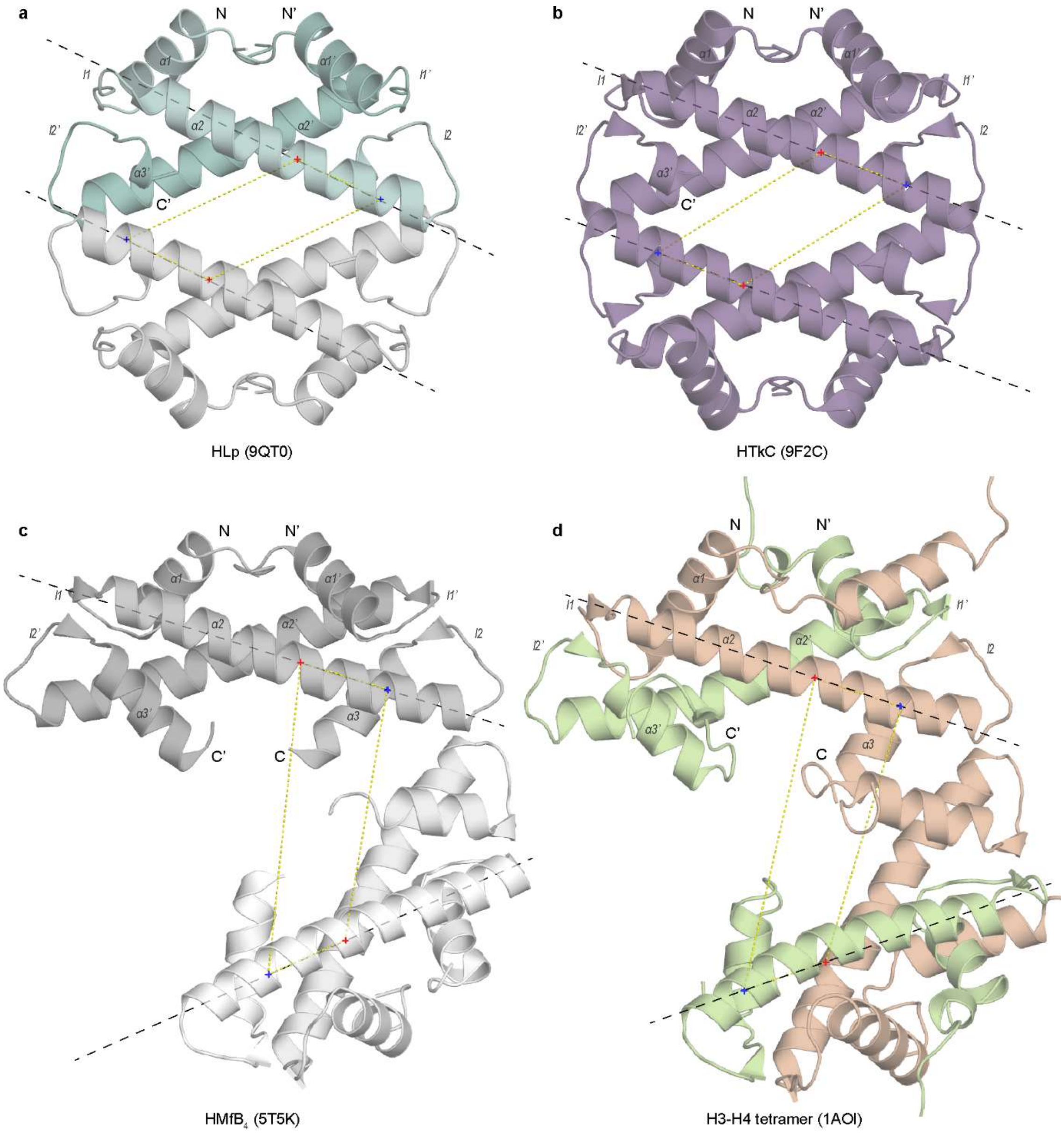

e

	HLp (9QT0)	HTkC (9F2C)	$HMfB_4$ (5T5K)	H3-H4 tetramer (1AOI)
Dimer-dimer angle (deg)	172.99	172.21	135.39	140.58

Supplementary Figure 7

Structural comparison of FtF histone tetramers with chromosomal histones. a Tetramer of FtF histone HLp (PDB: 9QT0). **b** Tetramer of FtF histone HTkC (PDB: 9F2C). **c** Archaeal nucleosomal histone HMfB (PDB: 5T5K). **d** Eukaryotic histones H3-H4 (PDB: 1AOI; H3 shown in orange, H4 in green). All structures are displayed as semi-transparent cartoon models. To quantify the relative orientation of the two dimers within each tetramer, two representative atoms (indicated with red and blue asterisks) were selected and connected to form a reference line (grey dashed line). **e** Table summarizing the calculated angles describing the relative orientation of the two dimers.

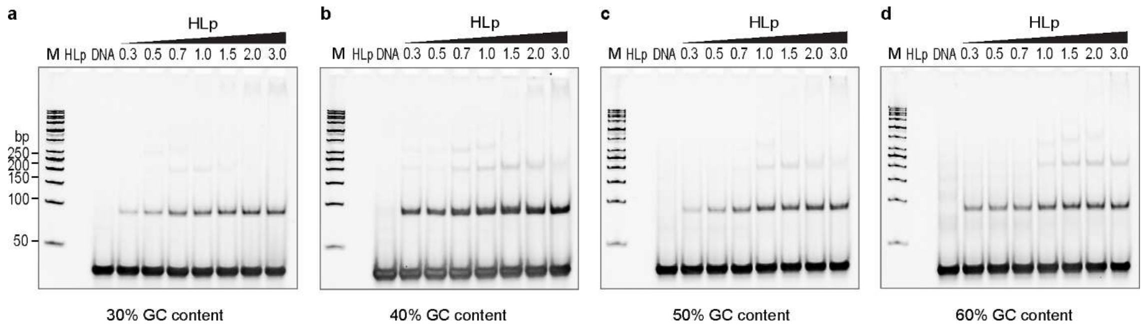

Supplementary Fig. 8

Binding of HLp to DNA fragments of variable GC content. EMSAs showing binding of HLp to DNA fragments 30-bp-GC30 (**a**), 30-bp-GC40 (**b**), 30-bp-GC50 (**c**), and 30-bp-GC60 (**d**). The molar protein to DNA ratios loaded in lanes 4–10 are indicated.

Original uncropped gel images are provided as a Source Data file.

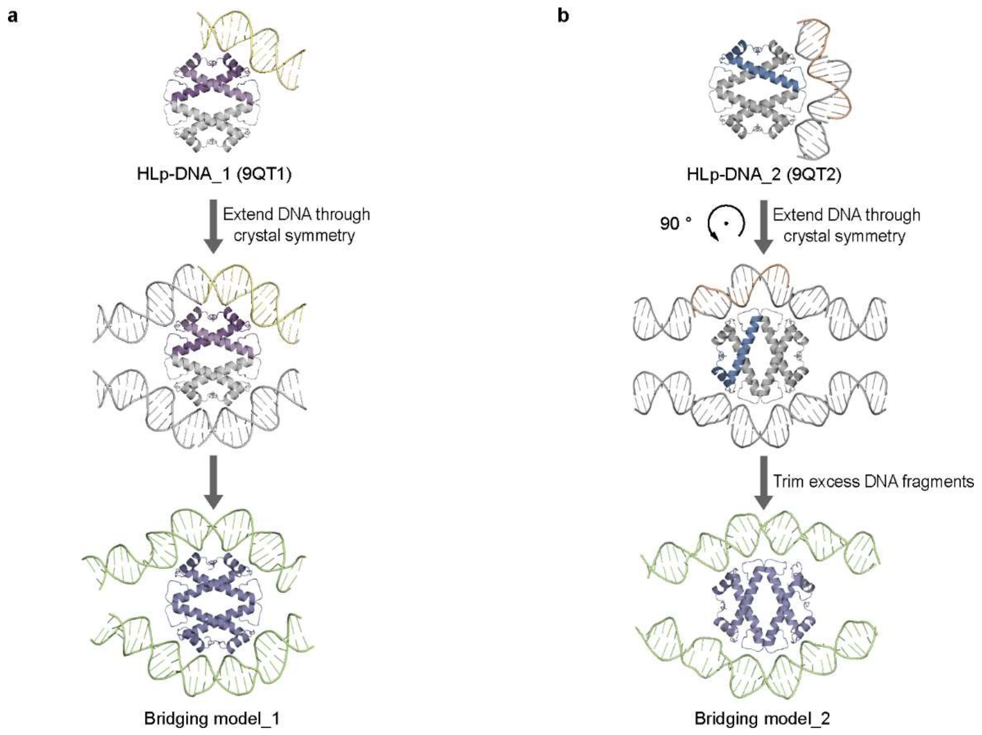

Supplementary Fig. 9

Workflow for generating starting models for the bridging mode. Shown are workflows used to construct the starting models: **a** bridging model_1 and **b** bridging model_2. The crystal structures of HLp-DNA_1 (PDB: 9QT1) and HLp-DNA_2 (PDB: 9QT2) are displayed with the contents of a single asymmetric unit highlighted in color and selected symmetry mates shown in gray. For improved visualization, the HLp-DNA_2 structure was rotated 180° along the z-axis before the DNA fragments were extended through crystal symmetry.

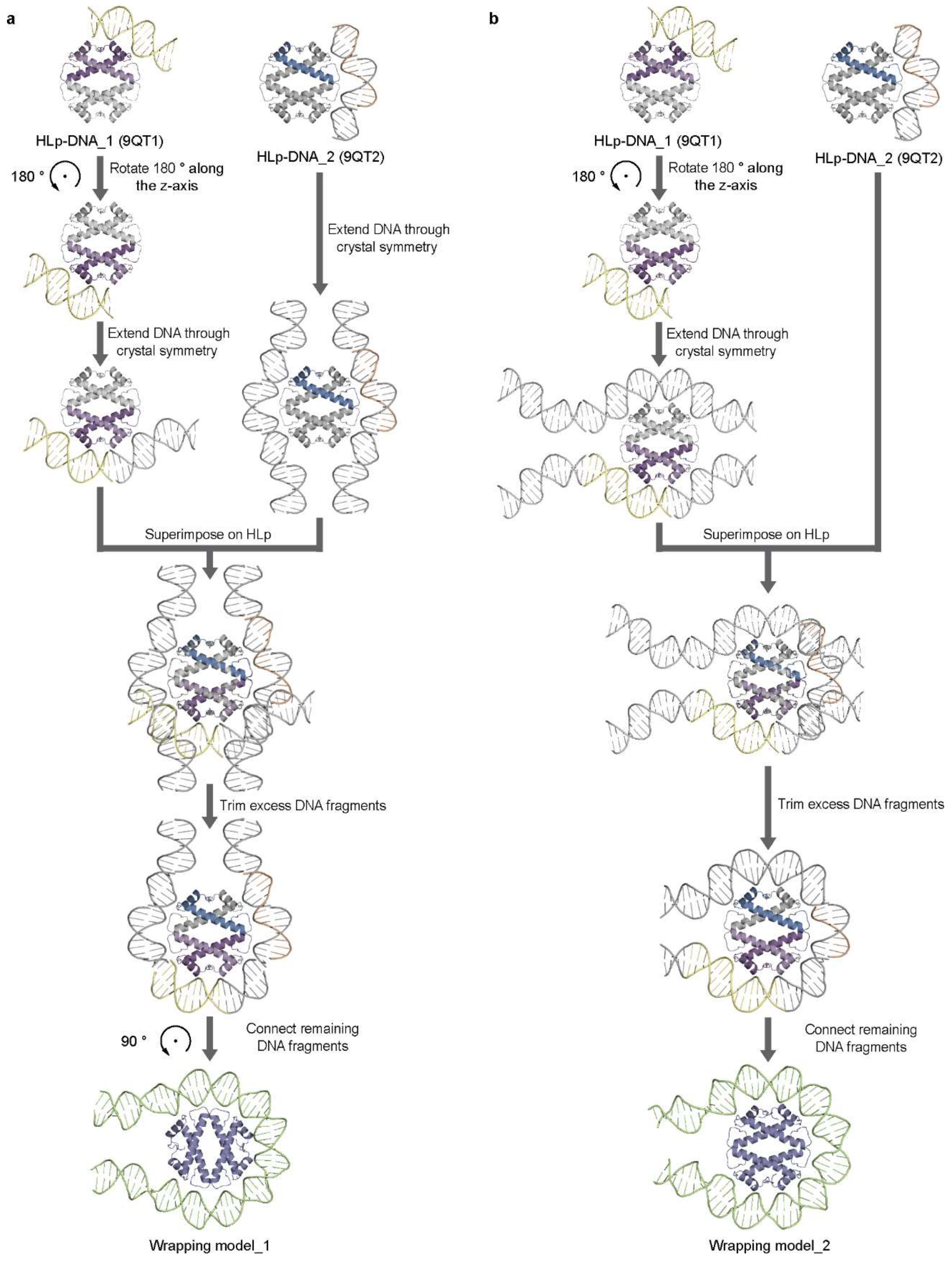

Supplementary Fig. 10

Workflow for generating starting models for the wrapping mode. Shown are the workflows used to construct the starting models: **a** wrapping model_1 and **b** wrapping model_2. The crystal structures of HLp-DNA_1 (PDB: 9QT1) and HLp-DNA_2 (PDB: 9QT2) are displayed, with the contents of a single asymmetric unit highlighted in color and selected symmetry mates shown in gray. For improved visualization, the HLp-DNA_1 structure was rotated 180° along the z-axis before the DNA fragments were extended through crystal symmetry.

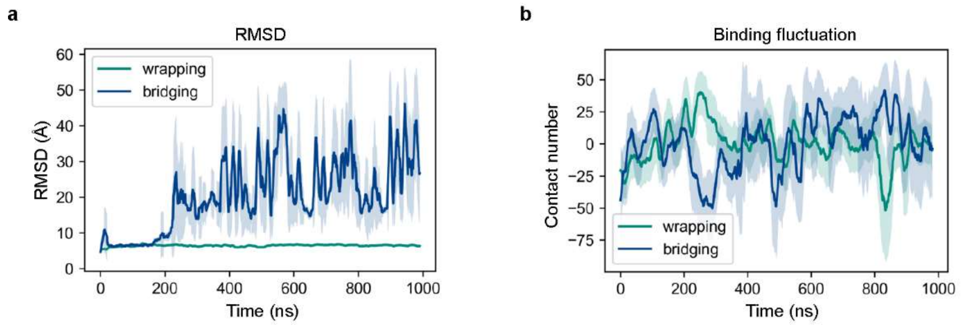

Supplementary Fig. 11
Overall characterization of the simulations of the two proposed binding modes for the replicates not selected for further analysis. a RMSD of the DNA backbones. **b** Binding fluctuation, defined as the deviation of contact number in each frame from the mean across all frames. Plots are smoothed with a window size of 100. Green and blue lines correspond to the wrapping and bridging models, respectively.

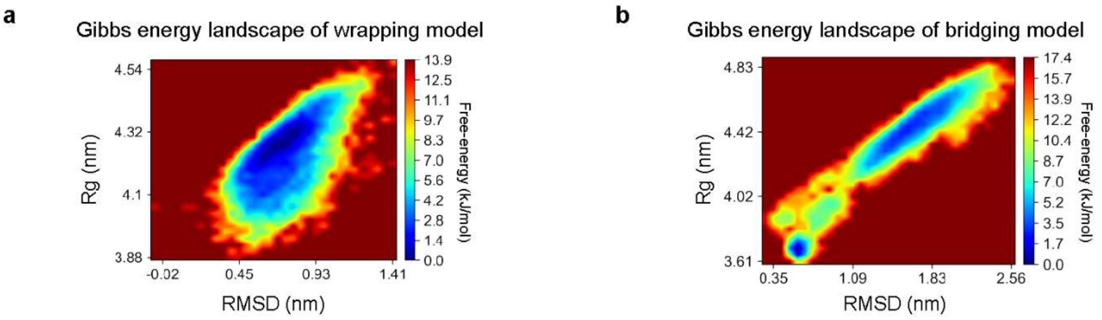

Supplementary Fig. 12

Free-energy landscapes of the two proposed binding modes. a Wrapping model. **b** Bridging model. The free-energy landscapes were constructed from simulation trajectories and projected onto two structural measures: the root-mean-square-deviation (RMSD) of the DNA relative to the reference structure and the DNA radius of gyration (Rg). The wrapping model samples a more compact region of the RMSD-Rg space, whereas the bridging model explores a broader distribution of conformations.

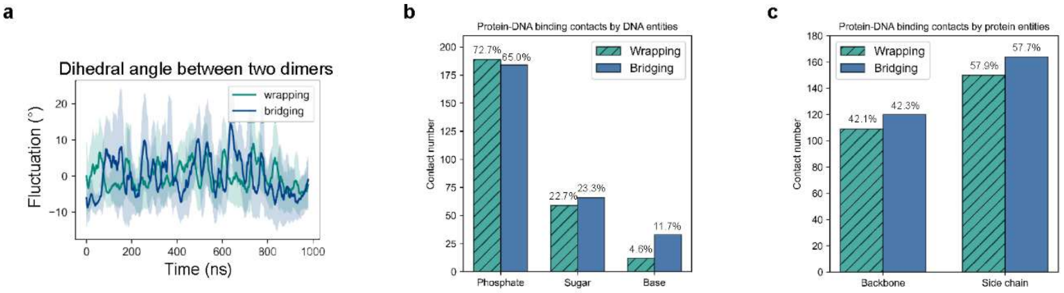

Supplementary Fig. 13

Dihedral angle fluctuations between dimers and protein-DNA interactions in the wrapping and the bridging models. a Fluctuation of the dihedral angle between two dimers over the course of the simulations. **b** Average number of protein-DNA atom-atom contacts categorized by DNA components (phosphate groups, sugars, and bases). **c** Average number of protein-DNA atom-atom contacts categorized by protein components (backbone and side chains). In all panels, green corresponds to the wrapping model and blue to the bridging model.

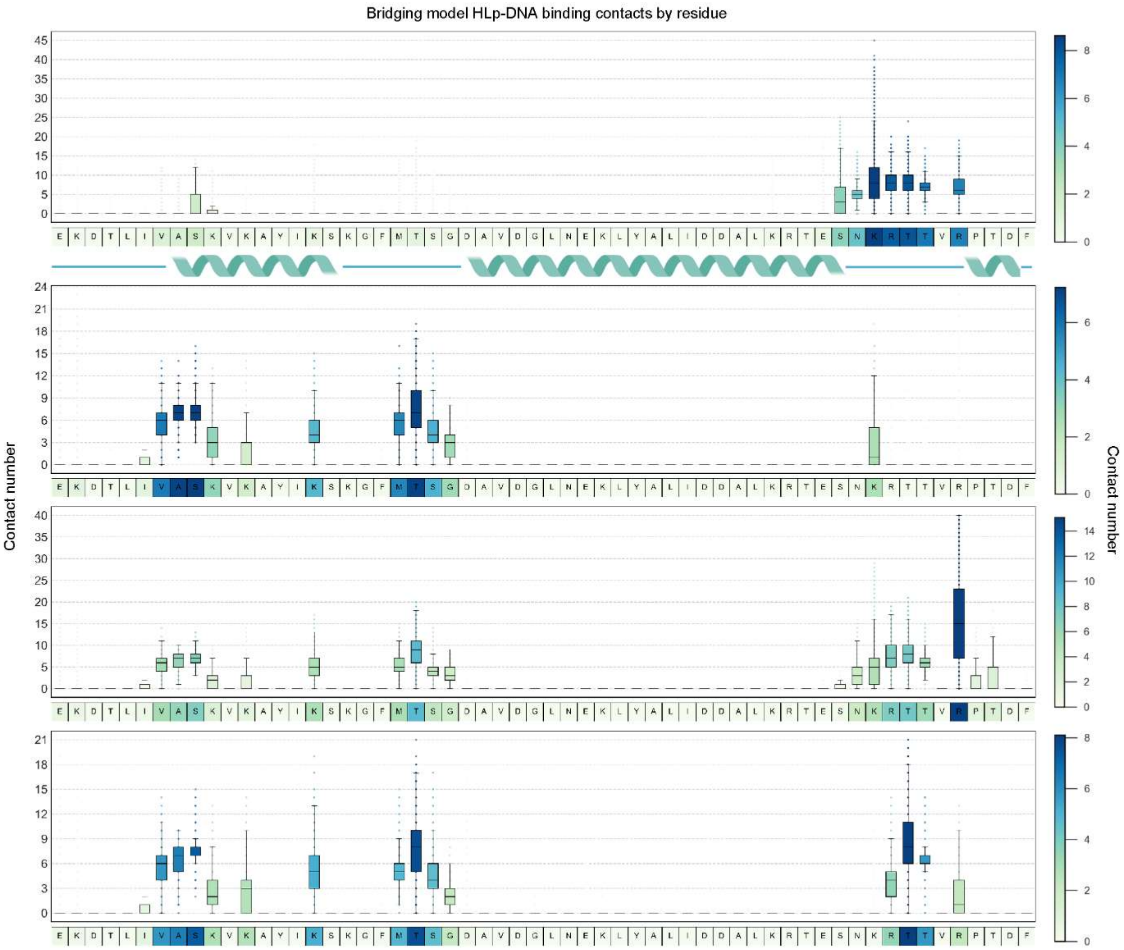

Supplementary Fig. 14

Residue-wise DNA binding contacts of HLp with DNA in the bridging model. Residue-wise boxplots showing the number of DNA contacts in the bridging model. Chains A, B, C, and D are displayed sequentially from top to bottom. Darker blue shades denote higher contact numbers. Center line indicates the median of contact numbers; box limits represent the 25th and 75th percentiles (Q1–Q3); whiskers extend to the most extreme data points within 1.5× the interquartile range (IQR) from Q1 and Q3. All individual contact values for each residue are plotted as semi-transparent dots, including outliers. Below each boxplot, the corresponding amino acid sequence is shown, with a secondary structure diagram provided beneath the first sequence for reference.

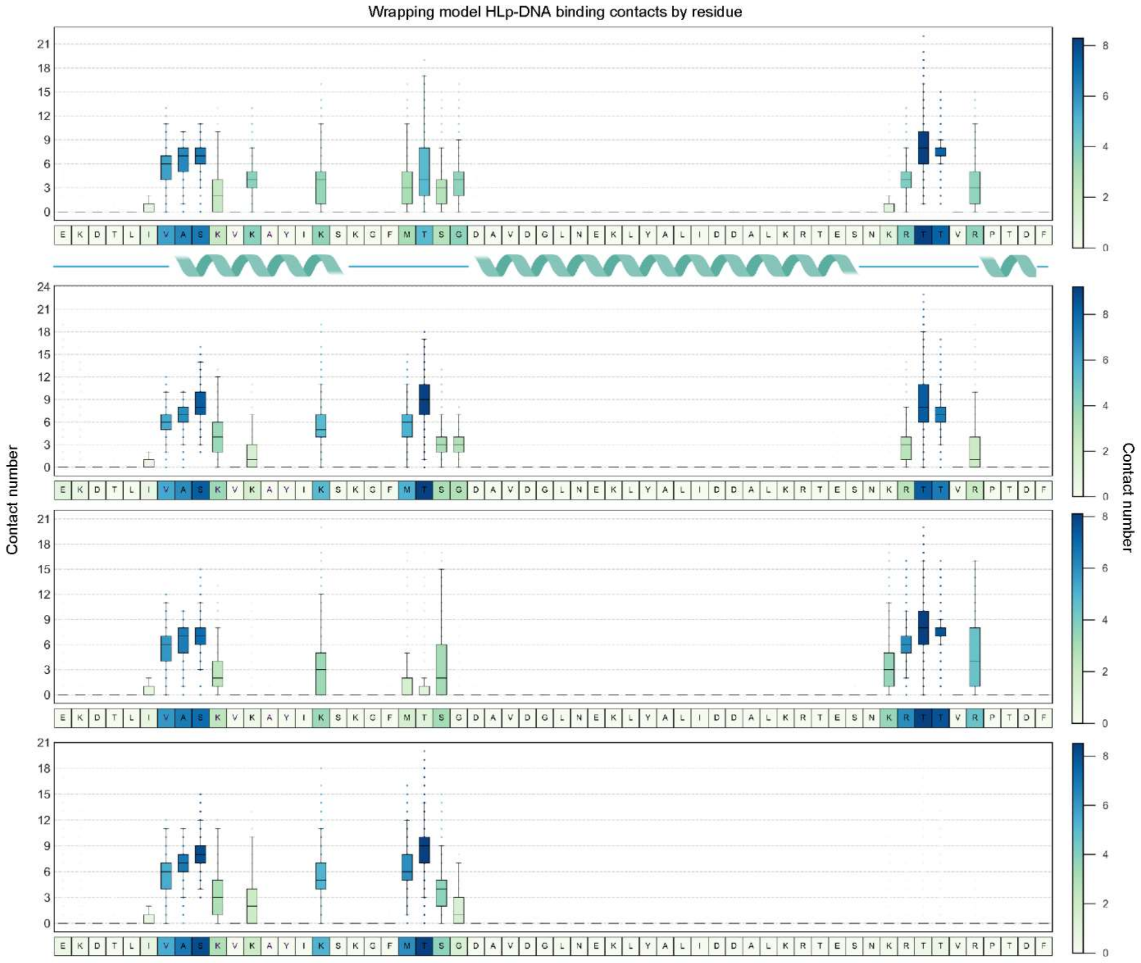

Supplementary Fig. 15

Residue-wise DNA binding contacts of HLp with DNA in the wrapping model. Residue-wise boxplots showing the number of DNA contacts in the wrapping model. Chains A, B, C, and D are displayed sequentially from top to bottom. Darker blue shades denote higher contact numbers. Center line indicates the median of contact numbers; box limits represent the 25th and 75th percentiles (Q1–Q3); whiskers extend to the most extreme data points within 1.5× the interquartile range (IQR) from Q1 and Q3. All individual contact values for each residue are plotted as semi-transparent dots, including outliers. Below each boxplot, the corresponding amino acid sequence is shown, with a secondary structure diagram provided beneath the first sequence for reference.

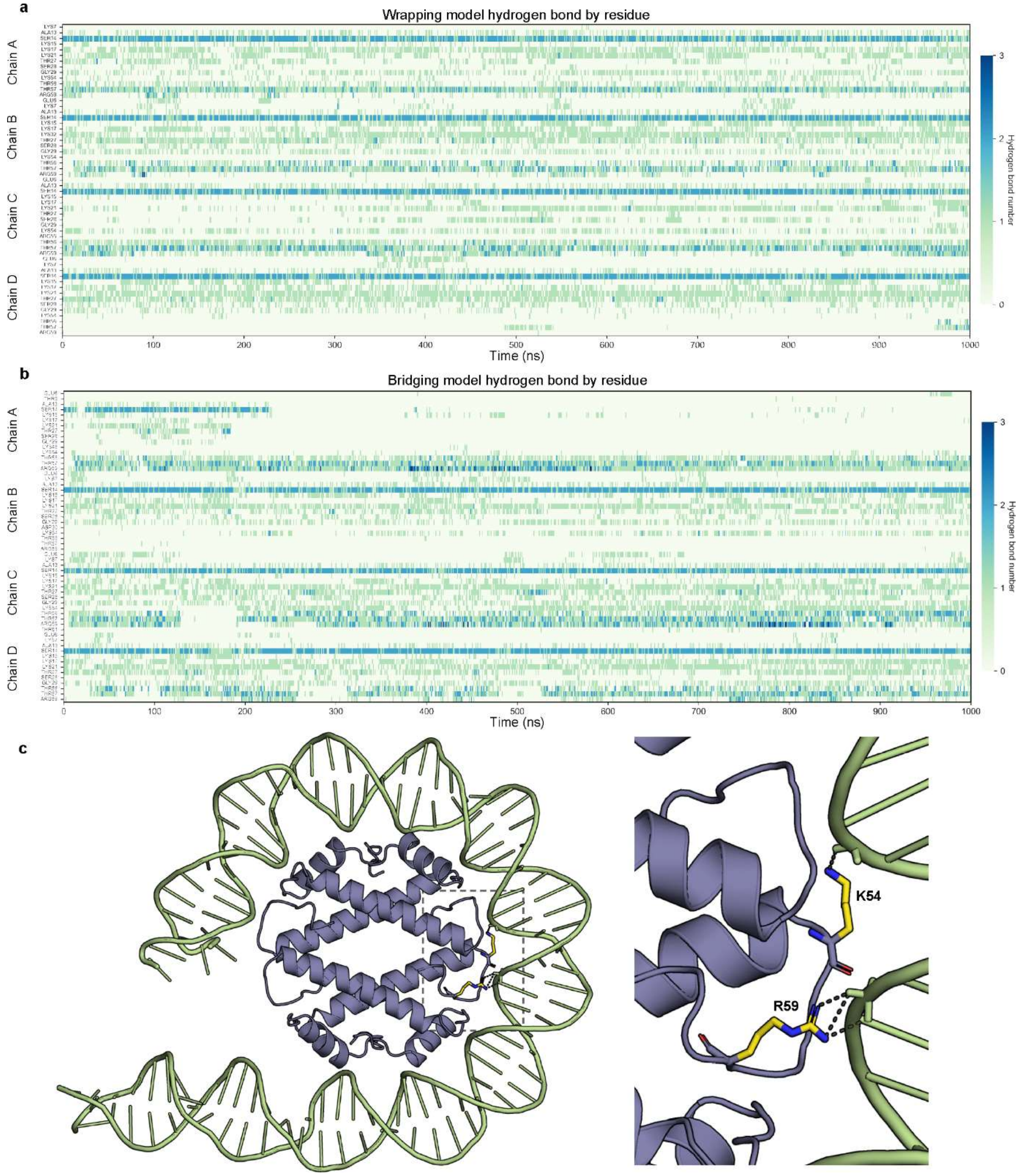

Supplementary Fig. 16

Protein-DNA hydrogen bond analysis of the wrapping and the bridging models. Heatmaps depicting the number of protein-DNA hydrogen bonds for each residue in the wrapping (**a**) and bridging model (**b**) throughout the simulation. The chain identifier of each residue is labeled on the left, with darker blue colors indicating a higher number of hydrogen bonds. **c** Cartoon representation highlighting structural details of residues K54 and R59 in one monomer of the wrapping model, captured at the 150 ns snapshot of the HLp-DNA simulation. Both residues are shown as sticks with yellow-colored side chains, and hydrogen bonds between residues and DNA are represented by dashed lines.

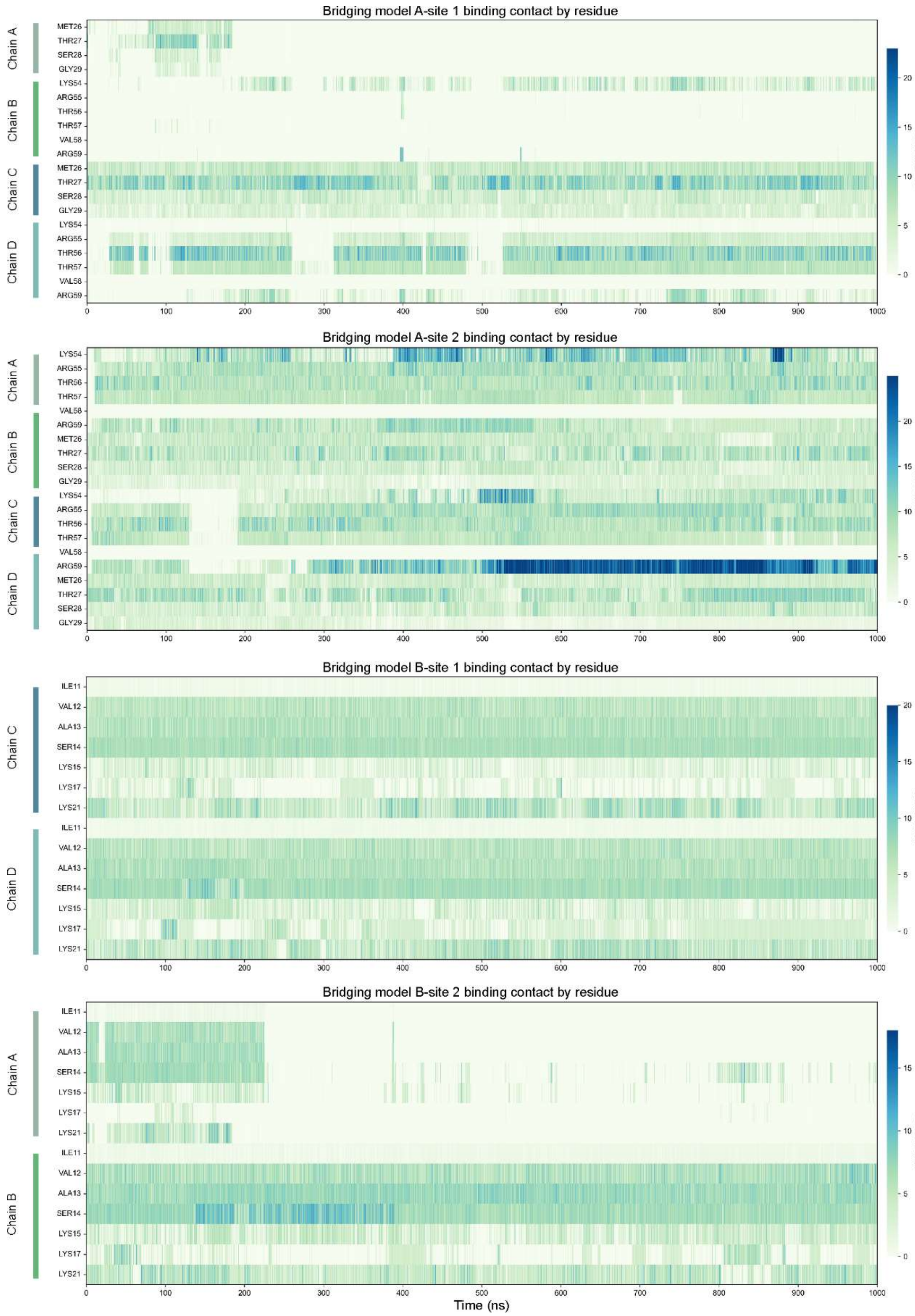

Supplementary Fig. 17

Protein-DNA contact analysis across binding sites in the bridging model. Heatmap illustrating the protein-DNA contact number for each residue across the four binding sites in the bridging model throughout the simulation. Darker blue colors indicate higher contact numbers. The four binding sites—A-site 1, A-site 2, B-site 1, and B-site 2—are arranged sequentially from top to bottom, with the chain identifier of each residue labeled on the left.

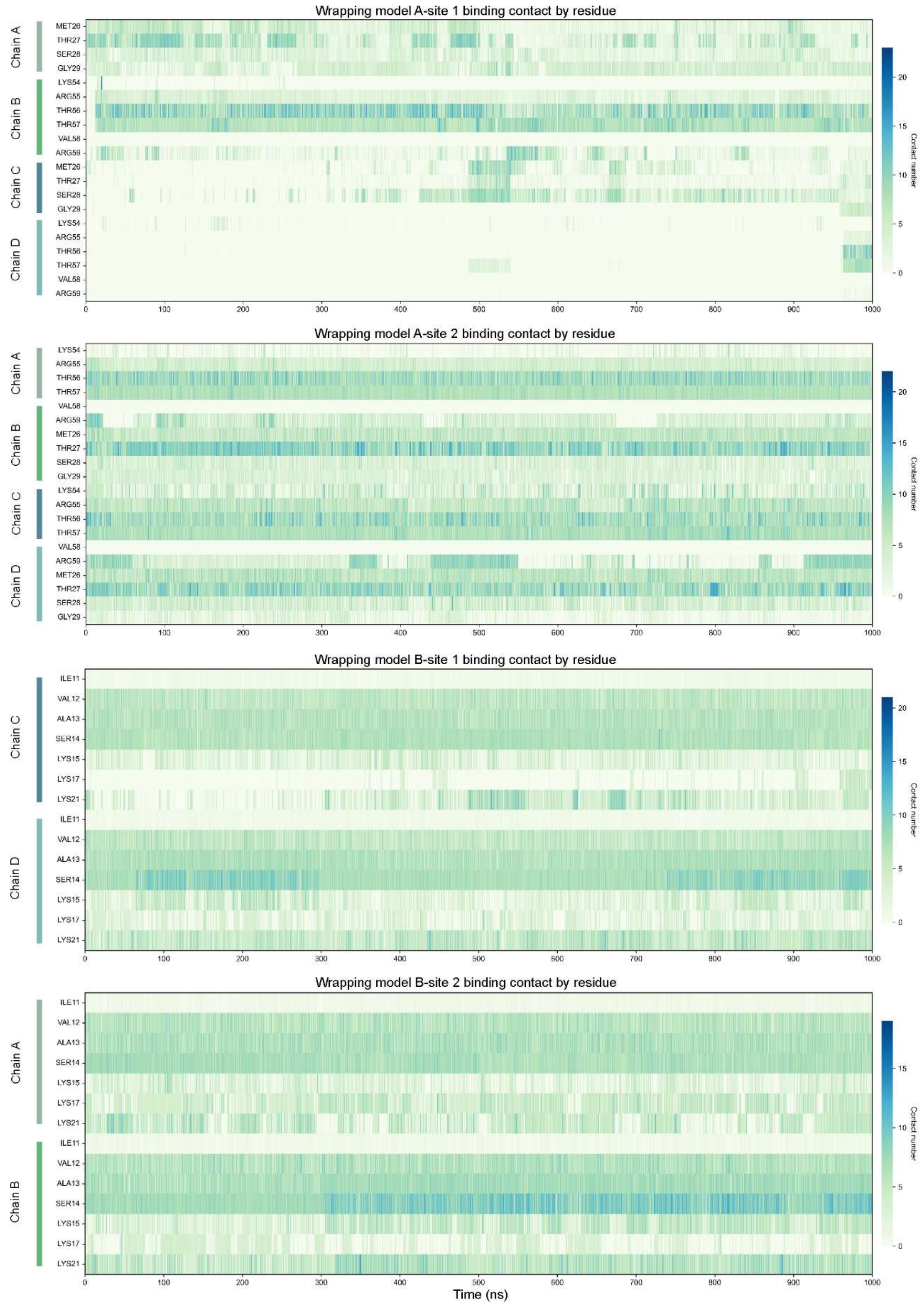

Supplementary Fig. 18

Protein-DNA contact analysis across binding sites in the wrapping model. Heatmap illustrating the protein-DNA contact number for each residue across the four binding sites in the bridging model throughout the simulation. Darker blue colors indicate higher contact numbers. The four binding sites—A-site 1, A-site 2, B-site 1, and B-site 2—are arranged sequentially from top to bottom, with the chain identifier of each residue labeled on the left.

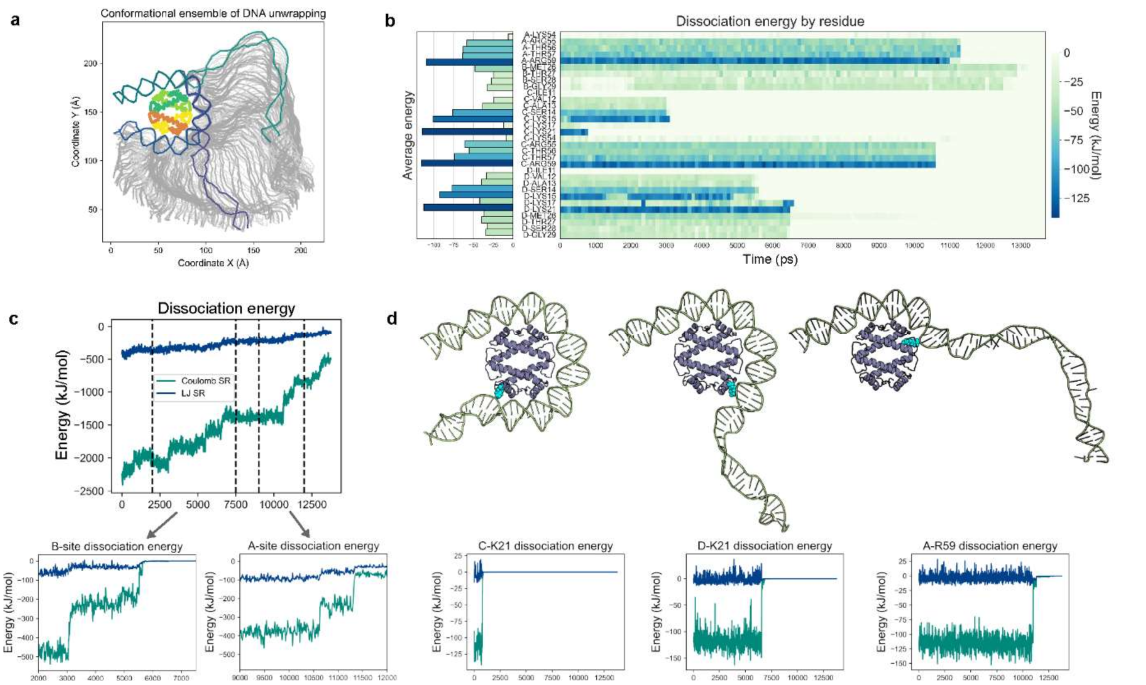

Supplementary Fig. 19

DNA unwrapping simulation. a Conformational ensemble of DNA throughout the simulation. The two ssDNA strands are colored light gray and dark gray, respectively. The HLp tetramer is colored by chains. **b** Heatmap illustrating the energy profile of each residue involved in A-site 2 and B-site 2 during the unwrapping simulation. Darker blue shades indicate lower energy. The average energy of each residue, excluding frames where the residue is dissociated (with an energy of 0 kJ/mol), is shown as a bar plot on the left. **c** Energy profile of the HLp-DNA system throughout the simulation, with blue and green lines representing short-range Lennard-Jones (LJ SR) potential energy and short-range Coulomb energy (Coulomb SR), respectively. The dissociation energy of the two binding sites involved in the simulation, B-site 2 and A-site 2, is presented below. **d** Dissociation energy profiles of three residues with low energy: Chain D-K15, Chain C-R59, and Chain B-K17. These residues are shown as spheres and colored in cyan.

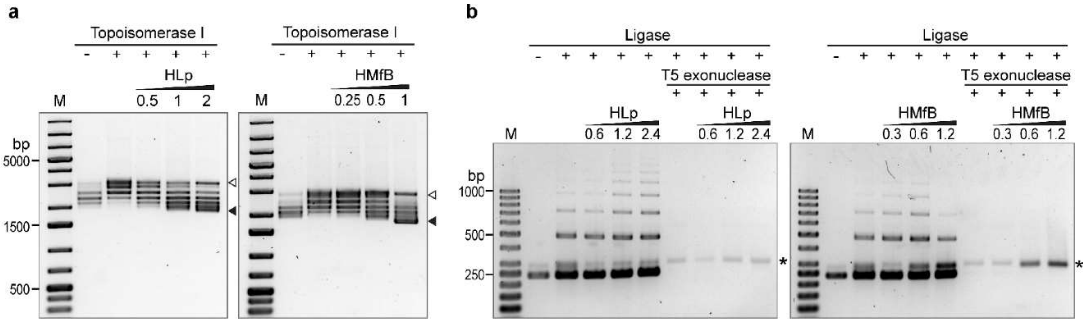

Supplementary Fig. 20

HLp binding changes DNA topology. a DNA topology assay with relaxed pUC19 plasmid DNA in the presence of HLp and HMfB. Protein to DNA mass ratios are indicated. The bands corresponding to relaxed (white triangle) and supercoiled pUC19 (black triangle) are labelled. **b** Ligase-mediated circularization assay with the 240-bp-GC40 DNA and HLp or HMfB. Samples are shown before and after T5 exonuclease digestion. The ratio of protein to DNA mass is labelled. Circularized monomeric DNA is marked with asterisks.

Experiments shown in (a) and (b) were performed in triplicate for HLp and in duplicate for HMfB. Original uncropped gel images are provided as a Source Data file.

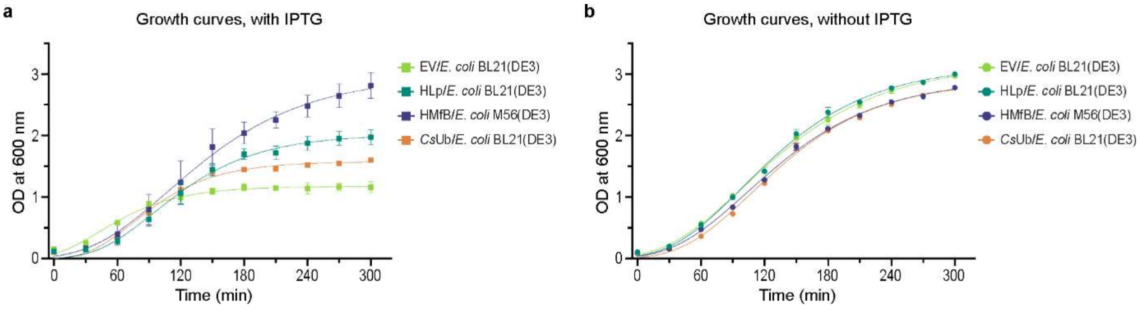

Supplementary Fig. 21

Growth curves of *E. coli* expression strains. *E. coli* strains were transformed with empty pET-30a(+) (EV), HLp in pET-30a(+), *Cs*Ub in pET-30a(+), and HMfB in pET-28a(+). Growth curves were recorded in triplicate under conditions with IPTG induction (**a**) and without induction (**b**). Source data are provided as a Source Data file.

Availability of Supplementary Data

Crystal structures have been deposited in the PDB under the corresponding accession codes.
Supplementary MD datasets are available on https://doi.org/10.5281/zenodo.15234989.
Source data for Supplementary Figures are provided with the accompanying Source Data file.

Supplementary References

1 Kabsch, W. Xds. *Acta Crystallogr D Biol Crystallogr* **66**, 125-132, doi:10.1107/S0907444909047337 (2010).

2 Tickle, I. J. *et al. STARANISO*. (Global Phasing Ltd, 2018).

3 Evans, R. *et al. Protein complex prediction with AlphaFold-Multimer*. (2021).

4 Jumper, J. *et al.* Highly accurate protein structure prediction with AlphaFold. *Nature* **596**, 583-589, doi:10.1038/s41586-021-03819-2 (2021).

5 Vagin, A. & Teplyakov, A. MOLREP: an automated program for molecular replacement. *Journal of Applied Crystallography* **30**, 1022-1025, doi:10.1107/s0021889897006766 (1997).

6 Emsley, P. & Cowtan, K. Coot: model-building tools for molecular graphics. *Acta Crystallogr D Biol Crystallogr* **60**, 2126-2132, doi:10.1107/S0907444904019158 (2004).

7 Murshudov, G. N. *et al.* REFMAC5 for the refinement of macromolecular crystal structures. *Acta Crystallographica Section D: Biological Crystallography* **67**, 355-367, doi:10.1107/s0907444911001314 (2011).

8 Van Der Spoel, D. *et al.* GROMACS: fast, flexible, and free. *Journal of Computational Chemistry* **26**, 1701-1718, doi:10.1002/jcc.20291 (2005).

9 Huang, J. *et al.* CHARMM36m: an improved force field for folded and intrinsically disordered proteins. *Nat Methods* **14**, 71-73, doi:10.1038/nmeth.4067 (2017).

10 Mark, P. & Nilsson, L. Structure and dynamics of the TIP3P, SPC, and SPC/E water models at 298 K. *The Journal of Physical Chemistry A* **105**, 9954-9960, doi:10.1021/jp003020w (2001).

11 Parrinello, M. & Rahman, A. Polymorphic Transitions in Single-Crystals - a New Molecular-Dynamics Method. *Journal of Applied Physics* **52**, 7182-7190, doi:10.1063/1.328693 (1981).

12 Bussi, G., Donadio, D. & Parrinello, M. Canonical sampling through velocity rescaling. *The Journal of Chemical Physics* **126**, 014101, doi:10.1063/1.2408420 (2007).

13 Darden, T., York, D. & Pedersen, L. Particle Mesh Ewald: An N·log(N) method for Ewald sums in large systems. *The Journal of Chemical Physics* **98**, 10089-10092, doi:10.1063/1.464397 (1993).

14 Hess, B., Bekker, H., Berendsen, H. J. C. & Fraaije, J. G. E. M. LINCS: A linear constraint solver for molecular simulations. *Journal of Computational Chemistry* **18**, 1463-1472, doi:10.1002/(sici)1096-987x(199709)18:12<1463::Aid-jcc4>3.0.Co;2-h (1997).

15 Hunter, J. D. Matplotlib: A 2D graphics environment. *Computing in Science & Engineering* **9**, 90-95, doi:10.1109/mcse.2007.55 (2007).

16 Van Rossum, G. & Drake, F. L. *Python 3 Reference Manual*. (CreateSpace, 2009).

17 Park, S. & Schulten, K. Calculating potentials of mean force from steered molecular dynamics simulations. *The Journal of Chemical Physics* **120**, 5946-5961, doi:10.1063/1.1651473 (2004).

18 Humphrey, W., Dalke, A. & Schulten, K. VMD: Visual molecular dynamics. *Journal of Molecular Graphics* **14**, 33-38, 27-38, doi:10.1016/0263-7855(96)00018-5 (1996).

19 Michaud-Agrawal, N., Denning, E. J., Woolf, T. B. & Beckstein, O. MDAnalysis: a toolkit for the analysis of molecular dynamics simulations. *Journal of Computational Chemistry* **32**, 2319-2327, doi:10.1002/jcc.21787 (2011).

20 Waskom, M. L. seaborn: statistical data visualization. *Journal of Open Source Software* **6**, 3021, doi:10.21105/joss.03021 (2021).

21 Bogomolovas, J., Simon, B., Sattler, M. & Stier, G. Screening of fusion partners for high yield expression and purification of bioactive viscotoxins. *Protein Expr Purif* **64**, 16-23, doi:10.1016/j.pep.2008.10.003 (2009).

22 Schindelin, J. *et al.* Fiji: an open-source platform for biological-image analysis. *Nature Methods* **9**, 676-682, doi:10.1038/nmeth.2019 (2012).

23 Baumgarten, T. *et al.* Isolation and characterization of the E. coli membrane protein production strain Mutant56(DE3). *Sci Rep* **7**, 45089, doi:10.1038/srep45089 (2017).

24 Rojec, M., Hocher, A., Stevens, K. M., Merkenschlager, M. & Warnecke, T. Chromatinization of Escherichia coli with archaeal histones. *Elife* **8**, doi:10.7554/eLife.49038 (2019).

25 Irwin, N. A. T. & Richards, T. A. Self-assembling viral histones are evolutionary intermediates between archaeal and eukaryotic nucleosomes. *Nature Microbiology* **9**, 1713-1724, doi:10.1038/s41564-024-01707-9 (2024).

26 Henneman, B., Heinsman, J., Battjes, J. & Dame, R. T. Quantitation of DNA-Binding Affinity Using Tethered Particle Motion. *Methods Mol Biol* **1837**, 257-275, doi:10.1007/978-1-4939-8675-0_14 (2018).
