# Supplementary material for: DNA Wrapping by a tetrameric bacterial histone
Source: Nat Commun. 2025 Dec 11;16:11108. doi: 10.1038/s41467-025-67425-w (PMC12701072; doi:10.1038/s41467-025-67425-w)
Supplement: Supplementary file 2 — Description of Additional Supplementary Files [file 41467_2025_67425_MOESM2_ESM.pdf]

Description of additional supplementary files

File name: Supplementary Code

Description: Python Script used to determine the dimer–dimer angle
