# Supplementary material for: DNA Wrapping by a tetrameric bacterial histone
Source: Nat Commun. 2025 Dec 11;16:11108. doi: 10.1038/s41467-025-67425-w (PMC12701072; doi:10.1038/s41467-025-67425-w)
Supplement: Supplementary file 3 — Supplementary Code [file 41467_2025_67425_MOESM3_ESM.pdf]

```python
from pymol import cmd, stored, CmdException
import numpy as np
import math
from chempy import cpv


def helix_orientation(selection, visualize=1, sigma_cutoff=1.5, quiet=0):
    """
    Calculate the orientation of a helix by averaging the direction of C(i)→O(i) bonds.

    Parameters:
    - selection: PyMOL atom selection string.
    - visualize: Show the orientation vector in PyMOL (1=yes, 0=no).
    - sigma_cutoff: Outlier removal threshold in standard deviations.
    - quiet: Suppress text output (1=yes, 0=no).
    """
    visualize, quiet = int(visualize), int(quiet)
    sigma_cutoff = float(sigma_cutoff)

    # Collect atom coordinates
    stored.coords = []
    cmd.iterate_state(1, selection + " and (name C+O)", "stored.coords.append([x,y,z])")

    if len(stored.coords) < 2:
        if not quiet:
            print("Error: Not enough atoms selected.")
        raise CmdException

    # Compute C→O vectors
    vectors = [np.array(stored.coords[i + 1]) - np.array(stored.coords[i])
               for i in range(0, len(stored.coords) - 1, 2)]
```

```
    # Initial mean vector
    mean_vector = np.mean(vectors, axis=0)
    mean_vector /= np.linalg.norm(mean_vector)

    # Remove outliers
    deviations = [np.dot(mean_vector, vec) for vec in
vectors]
    mean_dev, std_dev = np.mean(deviations),
np.std(deviations)
    filtered = [vec for vec, d in zip(vectors, deviations)
if abs(d - mean_dev) < sigma_cutoff * std_dev]

    if not filtered:
        if not quiet:
            print("Error: No vectors left after filtering.")
        raise CmdException

    # Refined mean vector
    mean_vector = np.mean(filtered, axis=0)
    mean_vector /= np.linalg.norm(mean_vector)

    # Helix center
    center = np.mean(stored.coords, axis=0)

    if visualize:
        cmd.pseudoatom("helix_center", pos=center.tolist(),
vdw=0.5, color="red")
        cmd.pseudoatom("helix_end", pos=(center +
mean_vector * 10).tolist(), vdw=0.5, color="blue")
        cmd.distance("helix_orientation", "helix_center",
"helix_end", label=0)
        cmd.show("sticks", "helix_center or helix_end")

    if not quiet:
        print("Helix orientation vector:", mean_vector)
        print("Helix center:", center)
```

```
    return center, mean_vector


def angle_between_helices(selection1, selection2,
visualize=1, quiet=0):
    """
    Calculate the angle between two helices using
helix_orientation.

    Example:
        select H1, chain A and resi 31-67
        select H2, chain B and resi 31-67
        angle_between_helices H1, H2
    """
    visualize, quiet = int(visualize), int(quiet)

    cen1, dir1 = helix_orientation(selection1, visualize,
quiet=1)
    cen2, dir2 = helix_orientation(selection2, visualize,
quiet=1)

    angle_rad = cpv.get_angle(dir1, dir2)
    angle_deg = math.degrees(angle_rad)

    if not quiet:
        print(f"Angle between helices: {angle_deg:.2f}°")

    if visualize:
        cmd.zoom(f"({selection1}) or ({selection2})",
buffer=2)

    return angle_deg


# Register commands in PyMOL
cmd.extend("helix_orientation", helix_orientation)
cmd.extend("angle_between_helices", angle_between_helices)
```
