# Supplementary material for: DNA Wrapping by a tetrameric bacterial histone
Source: Nat Commun. 2025 Dec 11;16:11108. doi: 10.1038/s41467-025-67425-w (PMC12701072; doi:10.1038/s41467-025-67425-w)
Supplement: Supplementary file 4 — Reporting Summary [file 41467_2025_67425_MOESM4_ESM.pdf]

nature portfolio

Corresponding author(s): Vikram Alva, Birte Hernandez Alvarez

Last updated by author(s): Oct 28, 2025

Reporting Summary

Nature Portfolio wishes to improve the reproducibility of the work that we publish. This form provides structure for consistency and transparency in reporting. For further information on Nature Portfolio policies, see our Editorial Policies and the Editorial Policy Checklist.

Statistics

For all statistical analyses, confirm that the following items are present in the figure legend, table legend, main text, or Methods section.

n/a	Confirmed	
☐	☒	The exact sample size (*n*) for each experimental group/condition, given as a discrete number and unit of measurement
☐	☒	A statement on whether measurements were taken from distinct samples or whether the same sample was measured repeatedly
☒	☐	The statistical test(s) used AND whether they are one- or two-sided<br>*Only common tests should be described solely by name; describe more complex techniques in the Methods section.*
☒	☐	A description of all covariates tested
☒	☐	A description of any assumptions or corrections, such as tests of normality and adjustment for multiple comparisons
☐	☒	A full description of the statistical parameters including central tendency (e.g. means) or other basic estimates (e.g. regression coefficient) AND variation (e.g. standard deviation) or associated estimates of uncertainty (e.g. confidence intervals)
☒	☐	For null hypothesis testing, the test statistic (e.g. *F*, *t*, *r*) with confidence intervals, effect sizes, degrees of freedom and *P* value noted<br>*Give P values as exact values whenever suitable.*
☒	☐	For Bayesian analysis, information on the choice of priors and Markov chain Monte Carlo settings
☒	☐	For hierarchical and complex designs, identification of the appropriate level for tests and full reporting of outcomes
☒	☐	Estimates of effect sizes (e.g. Cohen's *d*, Pearson's *r*), indicating how they were calculated

Our web collection on statistics for biologists contains articles on many of the points above.

Software and code

Policy information about availability of computer code

Data collection	Protein sequence homology analysis: BLAST (https://blast.ncbi.nlm.nih.gov/Blast.cgi), MMseqs2 (https://toolkit.tuebingen.mpg.de), CLANS (https://toolkit.tuebingen.mpg.de), EFI Genome Neighborhood Tool (https://efi.igb.illinois.edu/efi-gnt/);<br>Structure prediction: AlphaFold2 (AF2); CD spectroscopy: Spectra Manager 1.53 (JASCO), SEC–MALS: ASTRA v.7.3.0.18 (Wyatt Technology);<br>Imaging: Airyscan software, MST: MO Control V1.6 (NanoTemper Technologies)
Data analysis	Crystallography: XDS package 2024, STARANISO 2.4.19 (https://staraniso.globalphasing.org/cgi-bin/staraniso.cgi), MOLREP 11.7.02, Coot 0.9, REFMAC 5.8.0266;<br>Structure visualization: PyMOL 3.1.1;<br>Molecular Dynamics Simulation: GROMACS (version 2023.2), PDBFixer (version 1.10, https://github.com/openmm/pdbfixer), CHARMM all-atom force field (CHARMM36m), Python 3.8.5, VMD (version 1.9.4), MDAnalysis package (version 2.8.0), Matplotlib 3.6.2, Seaborn 0.12.2;<br>Image analysis: Image Lab 6.1 (Bio-Rad), FIJI (version 2.3.0);<br>Data plotting: GraphPad Prism 9

For manuscripts utilizing custom algorithms or software that are central to the research but not yet described in published literature, software must be made available to editors and reviewers. We strongly encourage code deposition in a community repository (e.g. GitHub). See the Nature Portfolio guidelines for submitting code & software for further information.

Data

Policy information about availability of data

All manuscripts must include a data availability statement. This statement should provide the following information, where applicable:

- Accession codes, unique identifiers, or web links for publicly available datasets
- A description of any restrictions on data availability
- For clinical datasets or third party data, please ensure that the statement adheres to our policy

Coordinates and structure factors of the crystal structures have been deposited in the PDB under entry numbers 9QT0 (DNA-free HLp), 9QT1 (HLp-DNA_1), and 9QT2 (HLp-DNA_2).
For the MD analysis, all configuration files, trajectory files, movies, and analysis scripts are deposited at Zenodo (https://zenodo.org/records/15234989).
The TPM data is deposited in the 4TU repository (https://data.4tu.nl) and accessible with the DOI 10.4121/5b604dd5-5498-46aa-b437-775e957f93e3.
Supplementary Information and Source Data are provided with this paper.

Research involving human participants, their data, or biological material

Policy information about studies with human participants or human data. See also policy information about sex, gender (identity/presentation), and sexual orientation and race, ethnicity and racism.

Reporting on sex and gender	n/a
Reporting on race, ethnicity, or other socially relevant groupings	n/a
Population characteristics	n/a
Recruitment	n/a
Ethics oversight	n/a

Note that full information on the approval of the study protocol must also be provided in the manuscript.

Field-specific reporting

Please select the one below that is the best fit for your research. If you are not sure, read the appropriate sections before making your selection.

☒ Life sciences ☐ Behavioural & social sciences ☐ Ecological, evolutionary & environmental sciences

For a reference copy of the document with all sections, see nature.com/documents/nr-reporting-summary-flat.pdf

Life sciences study design

All studies must disclose on these points even when the disclosure is negative.

Sample size	The sample sizes were selected in accordance with the available standard protocols, empirical values, and recommendations of the kit manufacturers.
Data exclusions	Molecular dynamics simulations were performed using two initial conformations for each model — DNA wrapping and DNA bridging. As the paired trajectories within each model showed highly consistent behavior, only one representative trajectory per model was selected for detailed analysis. According details are provided in the manuscript and Supplementary Information file.
Replication	All measurements (Microscale Thermophoresis, SEC-MALS, and Tethered Particle Motion) and electrophoretic analyses or assays (EMSA, MNase digestion, DNA topology assays) were performed in two or three replicates (as indicated) following the described protocols. Data from independent replicates showed consistent results. All corresponding source data, including replicates, are provided in the source data file.
Randomization	Randomization was not applicable to the assays and crystallographic structure determination used in this study. All experimental groups were defined by biochemical sample identity (purified protein constructs or DNA fragments) rather than individual biological subjects. For crystallography, MST, CD spectroscopy, SEC-MALS, EMSA, and tethered particle motion (TPM) assays, each sample condition represents a predetermined experimental group.
Blinding	Blinding was not applicable to the assays and crystallographic structure determination used in this study. All experimental groups were defined by biochemical sample identity (purified protein constructs or DNA fragments) .

Reporting for specific materials, systems and methods

We require information from authors about some types of materials, experimental systems and methods used in many studies. Here, indicate whether each material, system or method listed is relevant to your study. If you are not sure if a list item applies to your research, read the appropriate section before selecting a response.

Materials & experimental systems

n/a	Involved in the study
☒	☐ Antibodies
☒	☐ Eukaryotic cell lines
☒	☐ Palaeontology and archaeology
☒	☐ Animals and other organisms
☒	☐ Clinical data
☒	☐ Dual use research of concern
☒	☐ Plants

Methods

n/a	Involved in the study
☒	☐ ChIP-seq
☒	☐ Flow cytometry
☒	☐ MRI-based neuroimaging

Plants

Seed stocks	n/a
Novel plant genotypes	n/a
Authentication	n/a
